# Nasal Spritz

**Published:** 1871-04

**Authors:** Wm. Abram Love

**Affiliations:** Atlanta, Ga.


### NASAL SPRITZ.—*A New Apparatus for Treating Diseases of the Nasal Cavity—With Remarks.*

BY WM. ABRAM LOVE, M. D., Atlanta, Ga.

Read before the Atlanta Academy of Medicine.

:o:

In presenting the following paper to the Academy, it is done more for the purpose of calling the attention of its members, to a new and convenient form of Apparatus for treating diseases of the internal nasal cavity, and posterior naris, than with a view of entering into a discussion of the nomenclatures pathology, or treatment of the several diseases incident to this organ. The instrument is simple in its construction, and in my own hands, as well as in the hands of many members of the profession who have used it, has for many years proved invaluable; not only in the treatment of diseases of the nasal cavity but, *in arresting hæmorrhage from the nose*—in removing acrid, viscid secretions—in removing foreign bodies therefrom, and in treating wounds involving that cavity. Another, and great advantage is—that it can be improvised in a short time by any practitioner, with simply a wide mouth vial or bottle, a cork or two and a piece of india-rubber tubing or a gum-elastic catheter or bougie.

It consists of a vessel for holding the desired fluid, with an air-tight cap or stopple, through the latter are inserted two tubes—also air tight—the one inserted into the fluid to near the bottom of the bottle, to the utter end of this is attached an india-rubber tube six or eight inches long, terminating in a conical nozzle of proper size and shape to fill up, air tight, the external nasal orifice. The other tube passes through the cap or stopple—only, the lower end rests in the air-chamber at the top of the vessel, to the upper or outer end of this also, is attached an india-rubber tube, corresponding in length with the former and terminating in a mouth piece, around which the lips can be pressed air-tight.

To use the apparatus, the vessel is to be filled with any desired fluid, the nasal cone inserted into one side of the external nasal orifice with sufficient force to prevent regurgitation and with care not to close the orifice in its end by pressing it against the lining membrane. Then with a deep inhalation, the mouth piece is closed between the lips, and with a forcible exhalation, the air supplies the place of the fluid in the bottle, while the fluid is driven up through the nasal cone, passing in through the cavities of the nose, over the septum and down and out through the cavities on the opposite side of the *septum narium*, thus washing out, or applying medicated fluids to the whole of the Schneiderian membrane lining that passage.

I had invented and used this instrument long before seeing Doctor Thudicum's paper, or seeing his apparatus, and believe it to be in many particulars a better contrivance for the purposes for which the two instruments have been used. An allusion to these advantages here may not be out of place.

1st. The instrument presented is simple, small, convenient and portable, and can be constructed by any physician at short notice and little cost.

2d. In using Thudicums apparatus, the head must be thrown forward, the face downward, and even then, if the patient is not accustomed to its use, the fluid will often pass into the fauces and throat, often into the trachea, from failure to close the pharynx, or want of knowledge how to close it. While the instrument here presented, may be used by the patient, in a recumbent posture, or in any other position of the body, from the fact that *the pressure from within outward* CLOSING *the* pharynx, *in the act of forcing the fluid* through the nasal cavity, *is* GREATER, than the pressure *from without inward*, to open it, PLUS *the force* necessary to move forward the fluid used.

3d. The pressure and the necessary closing of the pharynx, in forcing the fluid forward, also closes the orifice of the Eustachean tubes leading to the inner ear, and prevents the ingress of the fluids into these tubes. This will often result from the use of *any other instrument* I have yet seen, particularly if the patient should swallow, or go through the motion of swallowing, during the passage of the fluids. This act, throwing open the Eustachean orifices while the force is *still applied*, must result in the introduction of the liquid into these tubes, from which evil, sometimes serious consequences, follow. In the apparatus here presented, the act of swallowing will cut off the applied force, and cause the flow to cease.

4th. By changing the nasal cone for the mouth piece and inveting the vessel (elevating the outer end of the longer tube above the level of the surface of the fluid,) this may be used for all the purposes for which Thudicum's instrument is used, and may be thus used where the patient cannot, from any cause, force the fluid through the nasal cavities by the act of exhalation, as in cases of wounds of the mouth, lips or cheeks, particularly in cases where they have been penetrated.

5th. It may be used for many purposes for which other instruments cannot. By attaching to the short or mouth tube an elastic syringe, medicated liquids may be injected through the nasal cavities or into other cavities, without throwing into them the least particle of air, where its introduction may not be desired, as in washing out the bladder or other organs, the air remaining in the chamber above.

6th. Substituting different sized nozzles, or spray points to the nasal tube, and a syringe to the mouth tube, it may be used where a steady stream, or fine spray is desired for washing wounds or orfices, as the eye or the ear. The elasticity of the air in the chamber above, while it regulates the force of the stream will deprive it of its *per sultum* character.

7th. The force used with this instrument is an *applied force*, and may be regulated at pleasure and independent of gravity. Where the tubes are too long, where they are too small or where the vessel is held too low, requiring an additional force to raise or propel the liquid used, it will not give the entire satisfaction that might be expected, when attention is given to these points.

The nasal or mouth piece can be constructed of hard or soft rubber, ivory, glass wood, or even cork, and should be constructed with a tube over which the india-rubber tubing may be slipped, that each patient may use his own mouth-piece and nozzle. They are better constructed of hard non-porous substances, to which will not adhere, matter, ephithelial scales, disintegrated, tissues or contaminated excreta, as by a want of this precaution, diseases of an infectious or contagious character, may be propagated, where different individuals use the same instrument.

For the purpose of clearing out the nasal cavities, and preserving the pituitary membrane in a healthy condition, where there exists an unhealthy tendency—this apparatus has given much saisfaction, while from its size and convenience of transportation and use, it has many advantages not presented in any other and for these reasons is commended to the favorable notice of the profession.

In case of *hœmorrhage from the nose*, whether *active or passive;* the use of the *per-salts* of iron, *per-sulphate* or *per-chloride* will effectually arrest the flow. Other styptics or astringents will often accomplish the same object. In many instances patients suffer from a predisposition to this form of hœmorrhage, and in some it will prove fatal unless proper aid is timely given. In such cases, armed with an instrument of this kind, and a solution of one of the hœmostatic salts of iron, a patient and his attending physician may feel much more at ease. Where hœmorrhage from the nose presents itself as a *vicarious* discharge, as is sometimes the case, it is obstinate in the extreme—plugging both the anterior and posterior nares will not avail—where the exhadation is the result of relaxation of tissues, or long continued chronic disease and nothing short of the coagulation of the bood in the very orifices of the exuding vessels themselves will arrest the discharge, we have the means here of bringing the remedies thoroughly and effectually in contact with every portion of the lining membrane of this irregular cavity.

With the excepion of *this one alarming disease*, *symptom* or *accident*, as the case may be, the physician is too seldom called to treat diseases of the nasal cavity until they have advanced to a chronic stage, or assumed a formidable character, when it is difficult to relieve them without more or less damage to the internal structure—the result of the long neglect of proper remedies.

The valuable time lost in dealing with or testing the efficacy of some worthless if not absolutely injurious quack nostrum, can be gained, if gained at all, only by long continued persistence in a proper plan of treatment.

The very anatomical construction of the internal naris, not only renders the cavity peculiarly susceptible to disease, but makes that disease, in many instances, spedily fatal to some portions of the bones and membranes covering the same.

The *bones* forming most of the boundary of the *upper*, *middle* and *lower meatuses* are exceedingly thin; their *periosteum* is in many places supplied with blood through the vessels of their covering mucous membrane. Any diseased action in this membrane, tending to obstruct the circulation in the periosteal membrane, cutting off the supply to the thin bony partitions, must of necessity, lead to *necrosis*.—This in turn increases the iritation and results in the opening of the adjacent cells. The decomposition of the bones, the retention within their cavities of disintegrated tissues, puss, mucous and other excreta—in a state of decomposition, not only emits an odor offensive in the extreme, but acts as a a continued source of irritation until the powers of the olfactory are blunted or its fillaments spreading over the pituitary membrane so damaged as to destroy entirely the sense of smell.

The disturbances leading to these results are in the outset trivial in their character—their onward progress slow and imperceptable to the patient. The offensiveness of a discharge is not in the commencement likely to attract attention, as under such circumstances, the sense of smell is impaired and the olfactory accustoms itself to the gradual change.—Hence the physician is seldom consulted until the disease has assumed a formidable character.

The disorders giving rise to these more serious consequenses, may be simple *nasal catarrh*, or an inflamation partaking of a specific character. This is particularly the case where there is either *syphalitic* taint or a *scrofulous* diathesis, while again they may have an *eczematous* a *herpetic* or a scorbutic origin, or complication. Gastro-intestinal irritation, may give rise to irritation in the pituitary membrane resulting in chronic inflammation, as worms in children of scrofulous dialthesis, may lead to organic disease in the Schneiderian membrane.

In all cases the cause and general condition of the patient should be looked after, and such constitutional treatment, as well as local applications, resorted to as each individual case may indicate.

In typhoid fever, and kindred diseases, there is often *epistaxis*, and *always* arrest of secretion from the mucous membrane ot the nasal passages. In the exanthemata—particularly scarlatina, more or less inflammation exists. Patients suffering under such circumstances will find much relief from cleansing the cavity with tepid water, salt and water or a solution of soda.

This attention to the condition of the nasal cavities, will not only add much to the comfort of pationts suffering from these diseases, but must aid in promoting recovery by allowing the air to pass into the lungs without being contaminated with the effluvia arising from decomposed and decomposing tissues and secretions lodged within these cavities.

It could not be expected that a man would long preserve a healthy condition of system, if he breathed incessantly an atmosphere tainted with the foul effluvia arising from decomposing animal matter—at the door of his house such an object would be pronounced a neusance and speedily burned or burried; *yet in the very portals of the lungs*, how often do we find just such an object, in the decomposed tissues, decomposed bone and exudations from foul ulcers in a state of decomposition, contaminating the air at every inhalation but to increase that contamination at every exhalation? Could we expect for a single moment that the lungs of such patients would long insure a healthy condition, or that the general system could resist continually these deleterious influences?

But the investigation of this question was not contemplated here.

In the outset of this paper—as stated,—a discussion of the nomenclature, pathology and treatment of the several diseases incident to nasal cavity was not contemplated; therefore, with a brief allusion to the more important remedial agents to be used with the apparatus described, it will be brought to a close.

In all washes (except when *cold is* used as *an agent*,) the temperature of the fluid should range from 100° to 110° F. (about or a little above blood heat.)

*Washes for Cleansing the Cavity.—Tepid water* alone, or with the addition of one drachm to one ounce, of *common salt to the pint*, according to the indications will answer well as a solvent to the viscid secretions, and stimulate the mucous membrane to action. The saline solution is less disagreeable than plain water alone, and will, as a general rule, be preferred by patients.

As a *disenfectant* and gentle stimulant to an ulcerated surface,—Potas.: permang.: grs. ij.—x to Aq. Oi. will be found an excellent as well as an agreeable wash. It serves to oxidize decomposing secretions, and is entirely devoid of odor. It acts by liberating oxygen in the form of ozone, and deposits a small amount of caustic potash and black oxide of of manganese. Where an *astringent* action is desired in conjunction, sulph. alumina, ʒ i—ij. may be added to this with good effect. Labarracques solution, ʒ ss to ʒ i to Oj sulphite of soda, (*sodæ sulphis*) ʒ i—ij to Oj. Potassa chloras ʒ i—ij to oj. Ammonia mur: ʒ i—ij to Oj. Soda bi carb: ʒ i to ij to Oi. Aqua calcis—and new milk, separately or combined in equal proportions will in many cases answer well, as soothing, cleansing or disinfectant washes.

Among the *astringents*, are *sulph. alumina* already alluded to:

Zinci sulph, grs, v—x to Oj.

Cupri sulph, grs, ij—v to Oj.

Plumbi acetas, grs, x—xx to Oj.

Argenti Ni ras, grs, v—xx to Oj.

Acid tannic, ʒ ss—ʒ i to Oj.

Tr ferri chl, ʒ ss to f ʒ i to Oj.

As *alteratives:*

Tr iodine (or co. solut.,) f ʒ ss to f ʒ i to Oj.

Hydrarg, Chl, cor, grs 1—ij to Oj.

Or, the *black*, or *yellow* wash, diluted one-half, may be used where indicated.

As an *anodyne*, Tinct. Opii f ʒ i—ij to Oj, or an equivalent of Morphia, may be used with good effect.

Perhaps as *stimulants* should be classed cologne, f ℥ ss—i to Oj, Spirits wine in the same proportion, and the various tinct ures, according to their several properties, may be used in the proportion of f ʒ i to f ℥ ss, or f ℥ i to Oj.

Among the *hæmostatics* stand first the *per-svlphate* and *per-chloride of iron*, of various degrees of strength, according to the indications or emergencies of the case; and to these may added ice or salt, or both, (to the per chloride.)

In this list may be placed also many of the mineral or vegetable astringents enumerated above.

In the list of *emollients* may be mentioned milk, the mucilaginous fluıds, as flax seed tea, sassafras pith, elm water, olive oil, &c.

Glycerine, from its affinity for water, tends to dry the mucous membrane, and by admixture with the mucous secretions bebomes gummy and disagreeable, as is perhaps to a greater degree tanno-glycerine and its solutions.

Carbolic acid and its solutions, though excellent as a disinfectant, is, because of the odor, disagreeable to most patients.

In the choice of remedies, the indications of each individual case must guide the practitioner on general principles, but in the treatment of any, or all, too much attention cannot be given to *cleanliness* of the parts; for unless this is maintained, the healing process cannot progress satisfactorily, especially in ulcerative forms of disease.

Due attention to constitutional derangements, and to complications tending to modify results, must be given at all times, as without this disappointment will follow any and every plan of purely local treatment.

